# An Uncommon Cause of Hip Pain Originating From the Iliopsoas Muscle: A Case Report

**DOI:** 10.1155/cro/1491509

**Published:** 2025-02-10

**Authors:** Mustafa Ozcan, Emre Acar, Efe Kemal Akdogan, Mehmet Erduran

**Affiliations:** ^1^Department of Orthopaedics and Traumatology, Private Gazi Hospital, Izmir, Turkey; ^2^Department of Orthopaedics and Traumatology, Dokuz Eylul University Hospital, Izmir, Turkey; ^3^Department of Orthopaedics and Traumatology, Cigli Research and Training Hospital, Izmir, Turkey

## Abstract

A 43-year-old man presented with chronic left hip pain that had increased in severity in the last week. Imaging studies discovered a mass in the iliopsoas muscle. The mass was excised, and pathological examination revealed pleomorphic rhabdomyosarcoma. The patient was followed up for 2 years and demonstrated no recurrence, with good clinical results. Clinicians should follow a systematic diagnostic approach involving a detailed medical history, a comprehensive physical examination, imaging studies, and laboratory tests to evaluate masses in the iliopsoas region, leading to timely diagnosis and appropriate management. Rhabdomyosarcoma is already a rare malignancy in adults. Therefore, having it in the iliopsoas compartment makes this case unique. To our knowledge, there was only one previously reported case of rhabdomyosarcoma involving the iliopsoas.

## 1. Introduction

The iliopsoas muscle compartment can be involved in many different disease processes, including infection, tumor, and hemorrhage. Patients may present with a wide variety of symptoms that are often nonspecific, resulting in a delayed diagnosis. The iliopsoas compartment consists of the psoas major, psoas minor, and iliacus muscles. The muscles in the iliopsoas compartment are covered by the iliopsoas fascia. The muscles function as flexors of the thigh and trunk, and as lateral flexors of the lower vertebral column [1]. The upper part of the psoas muscle passes under the arcuate ligament of the diaphragm; as such, it is a potential channel between the mediastinum and the upper thigh. The iliopsoas compartment often acts as a conduit for the spread of disease. Understanding this complex anatomy is important for the accurate diagnosis of lesions affecting the psoas muscle [1, 2].

Primary tumors of the iliopsoas muscles are rare. The iliopsoas muscle may be affected by primary tumors such as liposarcoma, fibrosarcoma, leiomyosarcoma, and hemangiopericytoma [2]. The most common types of primary malignancies that metastasize to the iliopsoas muscle are lymphoma, melanoma, and carcinoma originating from the cervix, ovary, stomach, lung, and breast. Tumor involvement of the iliopsoas muscle is most often secondary to the direct extension of an adjacent tumor. The magnetic resonance imaging (MRI) features of iliopsoas lesions have been previously reported, but the results are nonspecific [2].

Usually, rhabdomyosarcoma is seen in children and the most common subtypes are embryonal and alveolar [3]. Rhabdomyosarcoma is the most prevalent childhood soft tissue sarcoma, accounting for more than 50% of all soft tissue sarcomas in this age group [3, 4]. Conversely in adults, rhabdomyosarcoma is extremely uncommon. Soft tissue sarcomas account for less than 1% of all adult malignancies. Additionally, rhabdomyosarcoma causes nearly 3% of all soft tissue sarcomas [4, 5].

Because they originate from primitive mesenchymal cells, rhabdomyosarcoma may derive from any soft tissue in the body [6]. The most common primary sites are genitourinary (24%), parameningeal (16%), extremity (19%), and miscellaneous other sites (22%) [6, 7]. Unlike in childhood, the percentage of rhabdomyosarcoma in the extremities increases in adulthood [8]. The trunk, abdomen, retroperitoneum, and perineal region are less common primary sites [9].

Rhabdomyosarcoma is already a rare malignancy in adults. Therefore, having it in the iliopsoas compartment makes this case unique. To our knowledge, there was only one previously reported case of rhabdomyosarcoma involving the iliopsoas [10].

### 1.1. Patient Consent for Publication Statement

The patient was informed that data concerning the case would be submitted for publication, and consent was given.

## 2. Case Report

**Figure 1:**
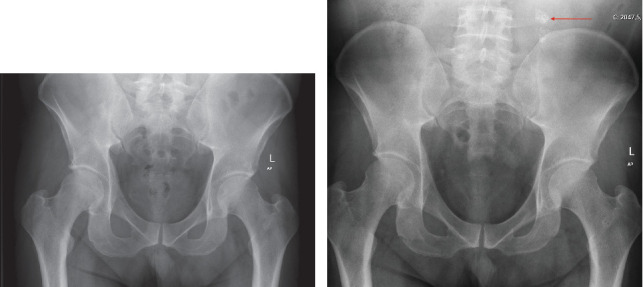
(a) Initial pelvic radiograph taken in the emergency room. (b) Second pelvic radiograph taken in the radiology department; red arrow showing the calcifications.

**Figure 2:**
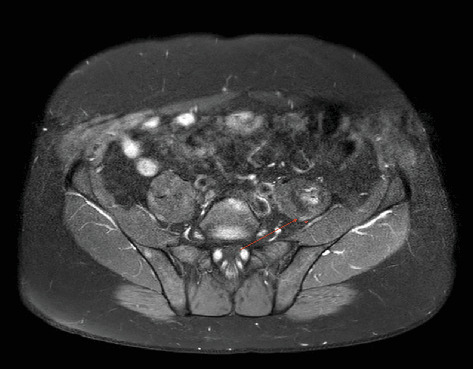
Mass originating from left psoas muscle. The red arrow showing the inferior border of the mass, asterisk showing the intersecting femoral nerve (axial T1-weighted MRI).

**Figure 3:**
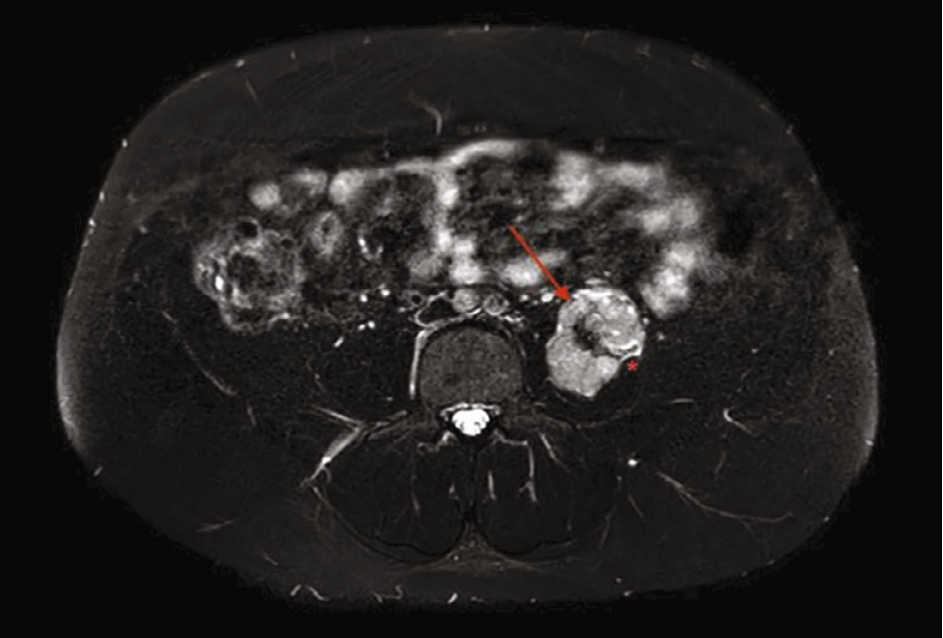
The red arrow showing lobulations and calcifications within the mass, asterisk showing the junction of tumor and psoas muscle (axial T2-weighted MRI).

**Figure 4:**
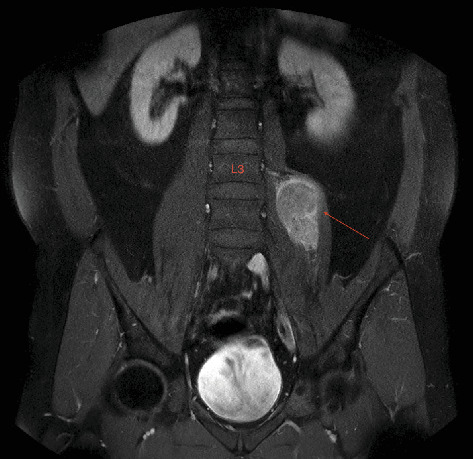
Mass lesion in the psoas muscle (red arrow) in which the upper border is seen as the third lumbar (L3) vertebra (coronal T1-weighted MRI).

**Figure 5:**
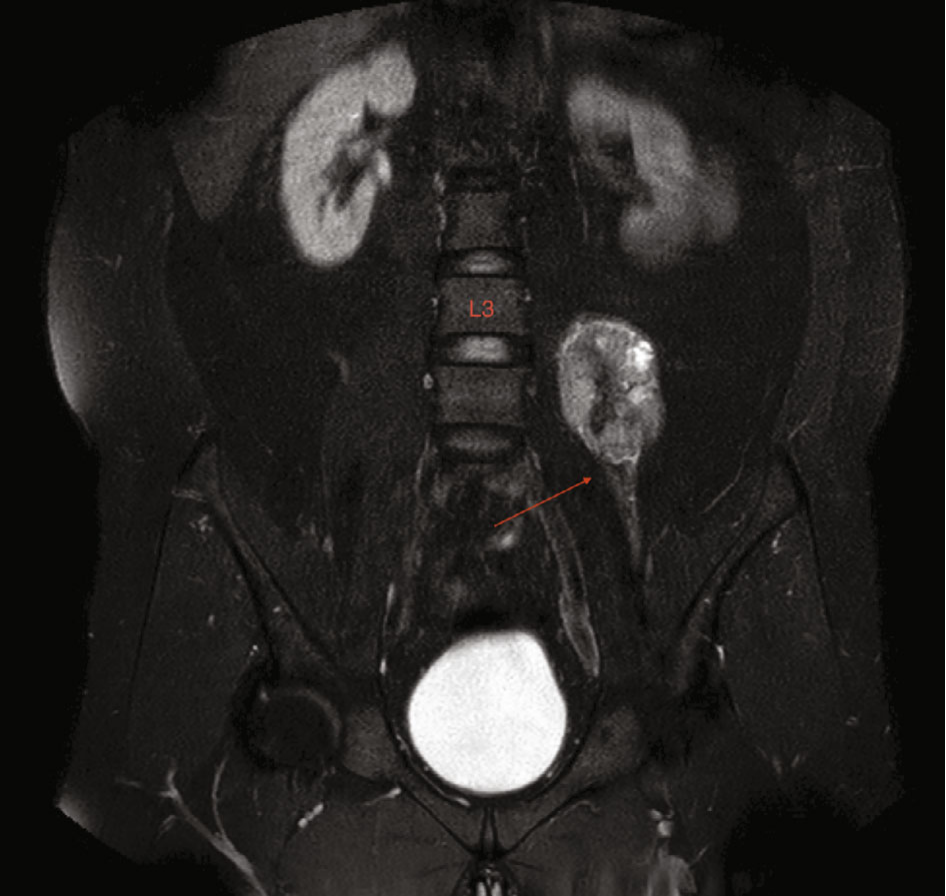
The red arrow showing the course of the femoral nerve intersecting the inferior border of the tumor. L3: third lumbar vertebra (coronal T2-weighted MRI).

A 43-year-old male was admitted to the emergency department with left hip pain. The pain, exacerbated by exercise and hip flexion, had been present for 1 year and had increased in intensity in the last week prior to admission. He had no history of trauma and had no comorbidities. His blood tests—complete blood count, erythrocyte sedimentation rate, C-reactive protein, electrolytes, liver function and kidney function tests—were all within normal range. Furthermore, the patient complained of malaise that had been ongoing for 2 months, loss of appetite, and a weight loss of 6 kg in the last 1 month. His physical examination demonstrated left hip pain that arose when the hip was in more than 40° of flexion, especially against resistance. He had no motor weakness in either extremity but described widespread numbness on his left lower extremity when his hip was in flexion. His Flexion Abduction External Rotation (FABER) test was positive. His gait was normal. Pelvic radiograph taken in the emergency room did not show any significant findings initially (Figure 1(a)). However, a thoracoabdominal computerized tomography (CT) scan revealed a mass lesion located posterior to the psoas muscle, which was noninvasive, contained amorphic calcifications, and was 64 × 29 mm in size. He was then discharged from the emergency department and was informed to refer to the department of orthopedics and traumatology the following day. Then, a subsequent pelvic radiograph revealed calcifications in the psoas muscle (Figure 1(b)). An abdominopelvic MRI was obtained by the orthopedics and traumatology department and demonstrated a massive lesion originating from the left iliopsoas muscle that extended along the surrounding fat tissue planes. Hypointense areas compatible with calcification in the T1-weighted series were seen. In the T2-weighted series, the mass was hyperintense, lobulated contoured, sharp-edged, markedly enhanced in postcontrast series, and with the dimensions of 72 × 45 × 50 mm (Figures 2, 3, 4, and 5).

After consultation, an iliopsoas muscle biopsy was performed by the interventional radiology department, in which the pathologic assessment revealed tumor cells consisting of fusiform and eosinophilic cytoplasm, showing rhabdoid morphology.

Once the decision was made concurrently with the follow-up examination for the surgical excision of the tumor, a preoperative MRI was obtained 2 months after the first MRI, which showed moderate expansion of the mass lesion, and a newly developed papillary extension component extending within the iliopsoas muscle.

### 2.1. Surgical Approach

**Figure 6:**
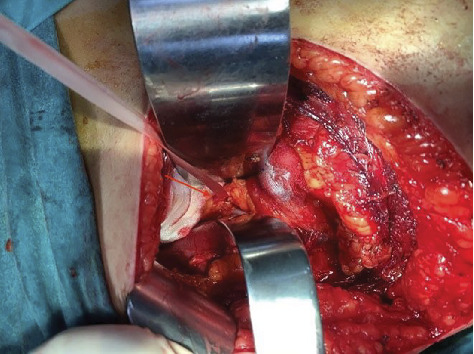
The adjacent ureter (arrow) was protected with the help of a tape.

**Figure 7:**
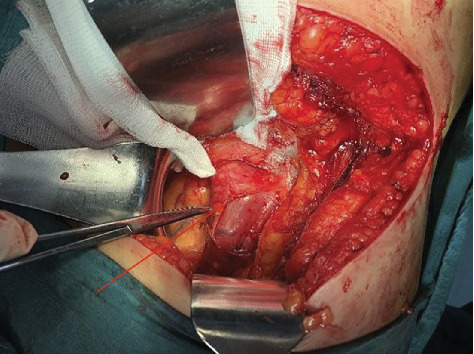
Mass originating from the left psoas muscle (arrow), pointed with the hemostat clamp.

**Figure 8:**
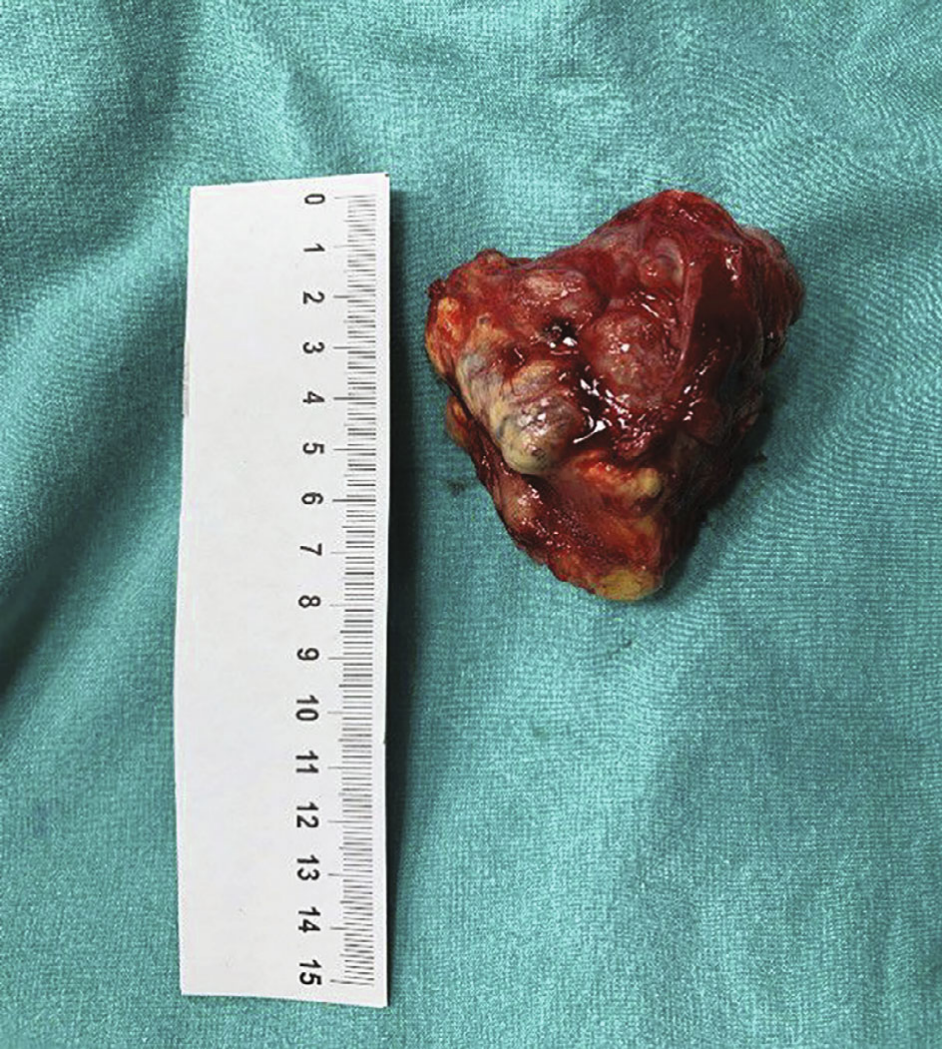
An approximately 8-cm mass was excised.

After placing the patient in the lateral decubitus position, a straight 10-cm transverse incision was made 2 cm below the 12th costa. The latissimus dorsi and serratus posterior inferior muscles were cut laterally, and external and internal oblique muscles were cut medially. The lumbodorsal fascia was incised, and the peritoneum was bluntly separated from the transverse abdominal muscle and retracted medially. After the transverse abdominal muscle was incised, the retroperitoneum was exposed. The ureter was observed adjacent to the left psoas muscle, and it was protected with the help of a tape (Figure 6). A mass approximately 8 cm in size was observed on the psoas muscle (Figure 7). It was excised from the psoas muscle “en bloc” by sharp and blunt dissection (Figure 8). Hemostasis was achieved and, after placing a drain, the incision was sutured appropriate to the anatomy. No early complications were observed. After histopathological evaluation of the mass, the diagnosis of “pleomorphic rhabdomyosarcoma” was made. The patient was followed up for 2 years and demonstrated no recurrence with good clinical results.

## 3. Discussion

Rhabdomyosarcoma must be differentiated from other tumors, especially Wilms tumor, Ewing sarcoma, neuroblastoma, liposarcoma, pheochromocytoma, osteosarcoma, acute myelocytic leukemia, acute lymphoblastic leukemia, and non-Hodgkin lymphoma [4]. Although the differential diagnosis of soft tissue sarcomas can be made radiologically among the diseases mentioned above, it is difficult to distinguish among soft tissue sarcomas themselves. The gold standard of diagnosis is usually tissue biopsy [4].

The most common pathologic subtype of rhabdomyosarcoma in adults is not otherwise specified (NOS) [4, 8]. NOS is a term to reflect a pathologist's inability to characterize the subtype. Pleomorphic rhabdomyosarcoma is the second most common subtype [8]. Both of these subtypes are much less frequent in children and young adults [8]. The pleomorphic subtype, an aggressive neoplasm, is more common in adults. It often occurs in the deep soft tissues of the extremities of patients older than 45 years [6, 8]. Pleomorphic rhabdomyosarcoma shows the most intense cytoplasmic eosinophilia. Tumor cells range from pleomorphic to epithelioid or spindle, and often abundant bright eosinophilic cytoplasm is observed [11, 12].

Psoas diseases include inflammation, hematoma, and tumors, and differentiating these three categories can be difficult, especially when there are no relevant clinical symptoms. Plain radiographs may not be sufficient if taken improperly like in this case. Generally, a psoas abscess comes to mind when a mass-like structure is seen in the psoas compartment on a CT scan. Psoas abscess rarely occurs in developed countries today. One of the most important reasons for this is the decrease in the frequency of tuberculosis. However, in developed countries, psoas abscesses may develop secondary to digestive system diseases [13]. In addition, the frequency of primary psoas abscesses has increased due to diabetes and immunodeficiencies. In their series, van den Berge et al. showed *S. aureus* as the most frequent pathogen in primary psoas abscesses [14]. In this case, the patient's laboratory findings did not suggest an infectious disease such as an abscess. In addition, radiological examinations performed after the CT scan indicated that the mass was not compatible with an abscess and had characteristics of malignancy.

Retroperitoneal tumors are difficult to diagnose because of the variations in pathology and the lack of specific radiological images [15]. Another diagnosis that consider after MRI findings exclude psoas abscess is benign schwannoma. Moreover, the literature review revealed case reports suggesting psoas abscess, but which emerged as benign schwannoma after drainage and biopsy [16, 17]. In our case, we decided to diverge from the diagnosis of schwannoma and plan an operation because of mass growth and suspicion of malignancy in the intermittent MRI findings.

Some of the patients with rhabdomyosarcoma could present with the complaint of mass without symptoms. The remaining patients may show signs and symptoms related to the primary tumor site or complications secondary to the tumor [4]. Pain is not usually a consistent symptom and often occurs because nerve compression in the area around the mass. In addition, the patient had numbness on his left lower extremity. This suggests that tumors in this area may dynamically compress the femoral and/or sciatic nerve in positions of hip flexion or rotation. Another possibility is the compression of the lumbosacral plexus and lumbosacral trunk. Hip joint disorders might cause pain in FABER or flexion, but numbness is unusual and might indicate a space-occupying mass [18]. In adults, swelling in areas such as the extremities is generally considered a musculoskeletal system injury and is often painless; therefore, the area in question may not be brought to medical attention for some time. The patient in this case complained of mild pain in his left hip, which increased with flexion. If radiological examinations are not performed and carefully investigated, the necessary treatment could be delayed. Therefore, differential diagnosis is essential when a patient presents with hip pain, but tests fail to explain the source of symptoms, or the patient worsens unexpectedly, or the source of pain cannot be attributed to a common etiology such as arthritis or muscle injury. These red flags may prompt the clinicians to obtain further tests for the atypical presentations of hip pain, and soft tissue sarcomas should always be considered.

## 4. Conclusion

When assessing longstanding hip pain, although degenerative processes make up most of the etiology, malignancy should be kept in mind in the differential diagnosis. To our knowledge, there was only one previously reported case of rhabdomyosarcoma involving the iliopsoas [10]. The fact that it is a rare and atypical cause of persistent hip pain makes it a more challenging diagnosis. Clinicians should raise suspicion when a patient worsens over time, as in this case.

## Data Availability

Previously reported data were used to support this study and are available in the references.
